# Analysis of the Impact of Configuration of the Stabilisation System for Femoral Diaphyseal Fractures on the State of Stresses and Displacements

**DOI:** 10.1155/2018/8150568

**Published:** 2018-01-08

**Authors:** Jakub J. Słowiński, Konrad Kudłacik

**Affiliations:** ^1^Department of Mechanics, Materials Science and Engineering, Faculty of Mechanical Engineering, Wroclaw University of Science and Technology, Wrocław, Poland; ^2^Orthopaedic and Trauma Department, Dr A. Sokołowski Specialist Hospital, Wałbrzych, Poland

## Abstract

**Introduction:**

The treatment of femoral diaphyseal fractures by intramedullary nailing has become a common procedure in orthopaedic surgery. The purpose of this numerical simulation was to present how the changes in configuration of the stabilisation system can affect the stress and displacement state in the bone tissue and implanted device.

**Material and Methods:**

The numerical comparison of the stabilisation variants for the type 32-A2 femoral diaphyseal fracture (according to the AO classification) performed by using the Charfix2 (ChM®) anatomical nail locked in a number of chosen ways. The displacement and the stress distributions both in the bone and implant were obtained and analysed by computational simulation.

**Results:**

In all models, there was the same characteristic distribution, which shows there were minimal rotational movements of the bone around the anatomical axis. In all cases, stress concentrations were generated in the nail material in the area of the fracture gap.

**Conclusions:**

The obtained results indicate that there is a visible advantage to one-plane distal stabilisation in the reduction of stresses regardless of the type of proximal stabilisation. The results of calculations indicate that the use of proximal stabilisation with a neck screw reduces the possibility of damage to the implant.

## 1. Introduction

Contemporary surgical treatment of fractures, termed stable osteosynthesis, consists of anatomical bone reduction and fixation of bone fragments, which prevents reciprocal displacement of bone fragments. The fixation is maintained until the bone union is achieved. Current designs enabled fixations thanks to which loads are transferred mostly by bone rather than fixation elements [1]. The choice of treatment for fractures depends on the age and general condition of the patient, the type of fracture, the competence of the doctor, and technical treatment options.

Stabilisation of femoral diaphyseal fractures in adults by intramedullary nailing is now the gold standard of treatment and a major advancement when compared with nonsurgical methods still used at the end of the 20th century, which were characterized by a high percentage of possible complications [1–5]. According to some new studies, stabilisation by means of locked plates provides a simpler, less invasive solution with fewer complications, which may be important for older osteoporotic patients, whose bones show osteoporotic features [6]. Standardization of fracture typology introduced by, among others, the AO Foundation, enables easier verification of the case and selection of the optimal method of obtaining bone union [7–10]. Literature cases of verification of commonly used classification systems suggest that those systems are not yet fully mature and, for very complex cases, the existing procedures are not completely effective [11, 12].

The correct placement of the implant together with its shape reflecting the curvatures of the femoral diaphysis allows for a more even transfer of loads and, therefore, diminishes the risk of occurrence of stress-shielding [13]. Appropriate insertion of the nail, followed by its securing with locking screws, provides stable fixation of bone fragments which, under dynamic loading conditions, enables regeneration of the bone tissue in the fracture gap.

Those methods still have a significant failure rate due to lack of bone union, which can be as high as 21% [14–17]. The relatively high percentage of failures in the form of nonunion of the bone contributes to numerical analyses because of the ability to compare several variants of stabilisation that are usually possible with the implant design. The capabilities of the numerical methods supported by clinical observations are now a powerful tool in predicting the effectiveness of treatment [1, 18].

The aim of the work was a numerical comparison of the stabilisation variants for the type 32-A2 femoral diaphyseal fracture (according to the AO classification) performed by using the Charfix2 (ChM) anatomical nail locked in a number of chosen ways. The obtained distributions of displacements and stresses in the bone-implant system were compared to each other and made it possible to determine which of the analysed types of stabilisation gave the most stable conditions for bone union.

## 2. Material and Methods

**Figure 1:**
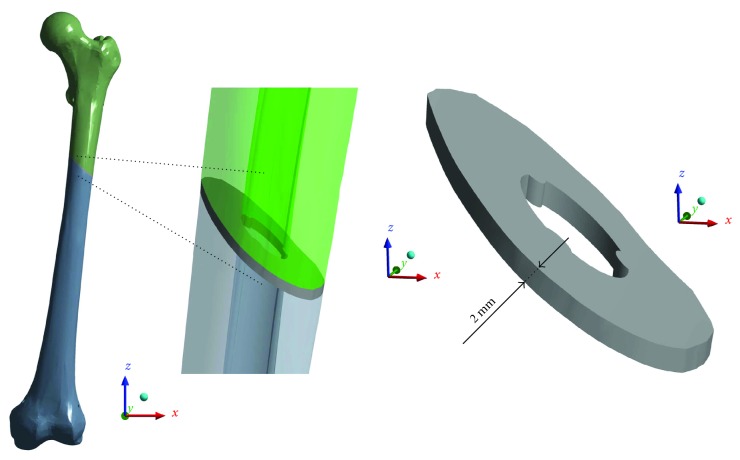
The geometric model of the femur and a fracture gap.

The geometric model of the femur was developed on the basis of computerized tomography of the bone of a healthy 45-year-old male and then imported into Ansys Workbench 17.2 software. Next, a 2 mm wide fracture gap was generated in the bone model (Figure 1). The size and the shape of the gap were determined by experimental and mathematical models [19, 20]. According to the classification maintained by the AO Foundation, the model reflected type 32-A2 fracture located in the middle of the femur length.

The nail model was developed based on the data from CT scans of a CHARFIX2 antegrade femoral intramedullary nail made by ChM. The anatomical nail, with a variable diameter and a length of 400 mm, was shaped in several planes to match the femoral diaphyseal anatomy. The main diameter of the nail was 11 mm, progressing in the upper part to the diameter of 14 mm. This size of the nail is typical for unreamed insertion. Static and dynamic holes were drilled in both the proximal and distal epiphysis. In the proximal part, the holes were located in the frontal plane, while in the distal part they were located in a number of planes, which gives a lot of freedom when choosing the nail locking method. The geometric design of the nail does not include any curves to simplify discretization of the model.

Matching screws for the selected model of the anatomical nail were also modelled in SolidWorks 2014. The length of the reconstruction screw was chosen based on the size of the femoral head, while the length of the locking screws was chosen depending on the femur diameter of the particular section. Screw models did not include thread outlines.

**Figure 2:**
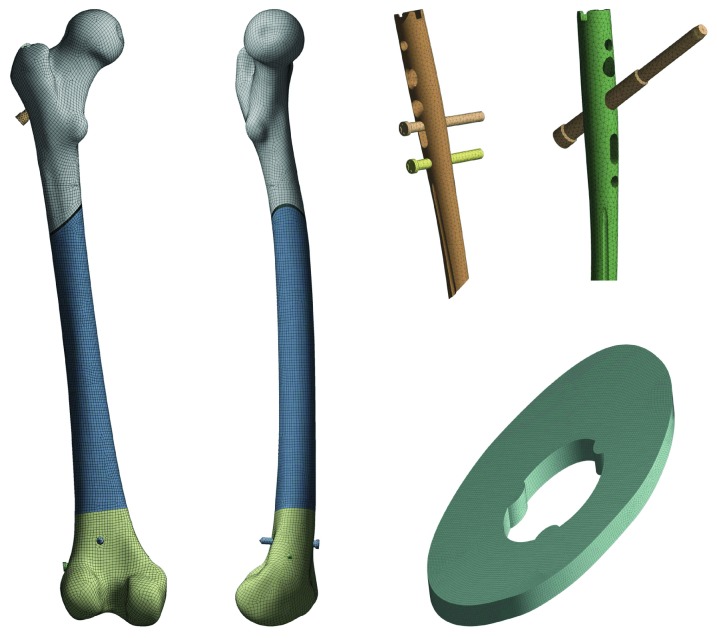
The finite element meshes of parts comprising the model: bone structure, implants (proximal parts), and fracture gap.

Discretization of the model was performed using higher-order 20-node elements for the model of the bone and fracture gap and 10-node elements for the model of the implant with bone screws. High-order elements were used because of the large differences between the material properties of the bone tissues and the fracture gap. The size of the element was determined to be 1.5 mm globally and 0.25 mm in the gap (Figure 2). The size and type of elements were selected based on a preliminary convergence analyses which have been performed on the bone model with unified material properties in order to check accuracy and numerical costs. This size represents a good trade-off between computational cost and numerical accuracy.

**Figure 3:**
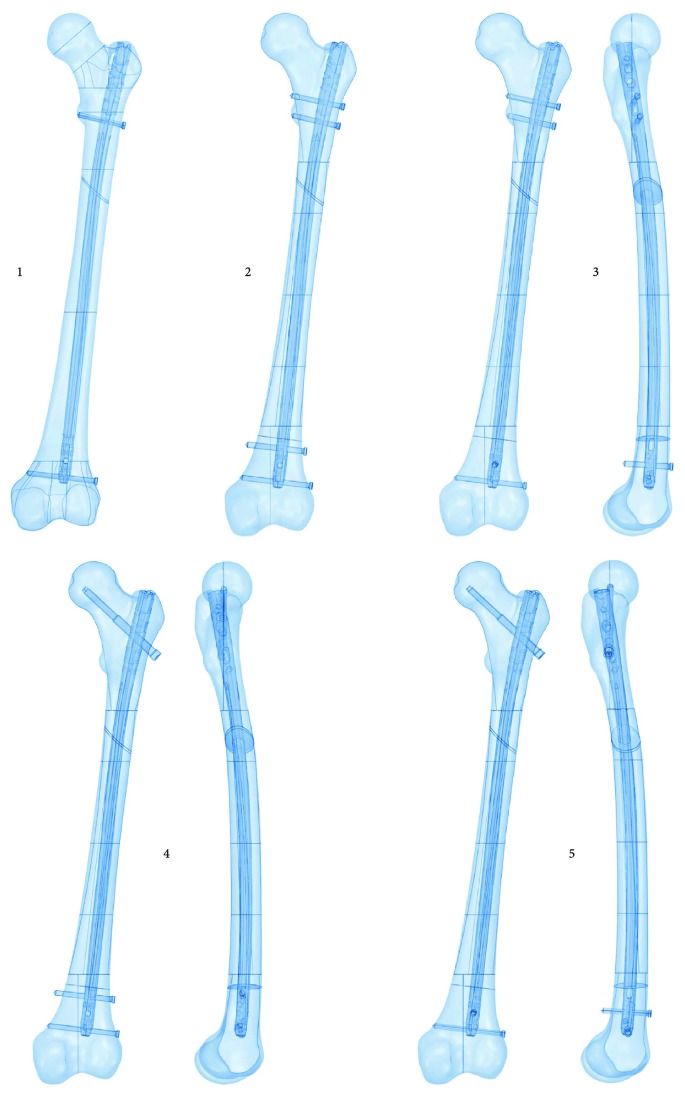
Analysed variants of intramedullary fixation.

The analysis covered five possible locking methods using both static and dynamic holes on one and two planes (Figure 3). The first way of locking used a static method with two locking screws in the frontal plane. One screw was inserted under the lesser trochanter, while the other screw was inserted in the last oval hole of the nail in the distal part, located above the level of the patellar surface and condyles.

In the second case, a compressive stabilisation method was used, that is, four locking screws were inserted in the frontal plane. In the proximal part, a screw was inserted in the oblong hole above the lower trochanter and a second screw was inserted in the oval hole below the lower trochanter. In the distal part, one screw was placed in the oblong hole and another one was placed below it in the last oval hole of the nail.

The third case involved the introduction of three locking screws in the frontal plane and—unusually for the femur—placement of one screw in the sagittal plane. In the proximal part, the screws were introduced in the same manner as in the second case. In the distal part, one screw was placed in the last oval hole of the nail in the frontal plane, while the second screw was placed above it in the sagittal plane.

The fourth way of locking used a reconstruction method. However, instead of the contemporary technique involving insertion of two screws, only one reconstruction screw was placed at an angle in the head of the femur. In the distal part, one screw was inserted in the oblong hole and another one was placed below it in the last oval hole of the nail.

In the fifth case, as in the fourth case, one reconstruction screw was used. In the distal part, one screw was placed in the last oval hole of the nail in the frontal plane, while the second screw was placed above it in the sagittal plane.

### 2.1. Loading Model

**Figure 4:**
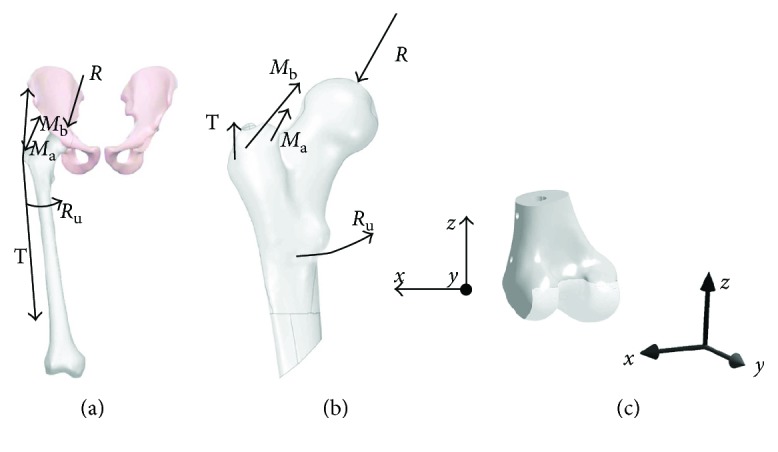
The boundary conditions: scheme of the Bedzinski's model (a), loading forces (b), and fixed support (c).

The developed numerical model was loaded in accordance with the loading model developed by Będziński at the Division of Biomedical Engineering and Experimental Mechanics of the Wrocław University of Science and Technology (Figure 4(a)) [21, 22]. In that model of hip joint, based on the ideas of Pauwels and Maquet, the effect of the internal rotators (*R*_u_), causing the rotation of the femur, was additionally taken into account. The following forces (Figure 4(b)) were found to be acting on the proximal femoral epiphysis during one-leg stance:

- Load from the trunk mass acting on the femoral head (*R*)
- Force of abductor muscles (*M*_a_) (*gluteus minimus*) and (*M*_b_) (*gluteus medius*)
- Force of the iliotibial band (*T*) (*tractus iliotibialis*)
- Force of thigh rotator muscles *R*_u_ (*iliopsoas*)

It was assumed that the force values will correspond to 50% of the full load for one-leg stance, reflecting saving of the limb by the patient (one-leg stance with support) (Table 1).

The fixation of the model was achieved by removing all degrees of freedom from surface knots within condyles, intercondylar fossa, and the patellar surface (Figure 4(c)).

The material properties were given in accordance with the data from Table 2 assuming that all objects are isotropic and linear. In order to more accurately reflect the bone-implant system, a distinction was made in the bone model between the compact and spongy bone tissues as well as the material in the fracture gap. In each case, a system of a physiologically normal, fractured, and stabilised bone was modelled. Thickness of the compact tissue was determined on the basis of medical records and CT images. CT scans were processed with 3DSlicer—an open source software for visualization and medical image computing. Then, for the given bone fragment, an appropriate number of layers of elements were selected to give them specific material properties. The implant used for the analysis had the parameters of a titanium alloy with an addition of niobium (Ti6Al7Nb), one of the typical materials used in the production of implants.

The next step was to define the type of contact between the given volumes. Although there are some minimal nail displacements inside the bone in the biomechanical system, no frictional contact was used for the point of contact between the bone and the implant; instead, bonded contact was used between all volumes. This allowed us to focus only on the issue of stabilisation of bone fragments.

The following data were recorded as a part of the conducted static analysis for the five cases of stabilisation of femoral fractures:

- Nodal displacements in the bone model (for each axis of the reference system), with particular emphasis on the place of application of the force
- Stress in the bone model at the point of contact with the implant
- Stress in the intramedullary nail model

## 3. Results

**Figure 5:**
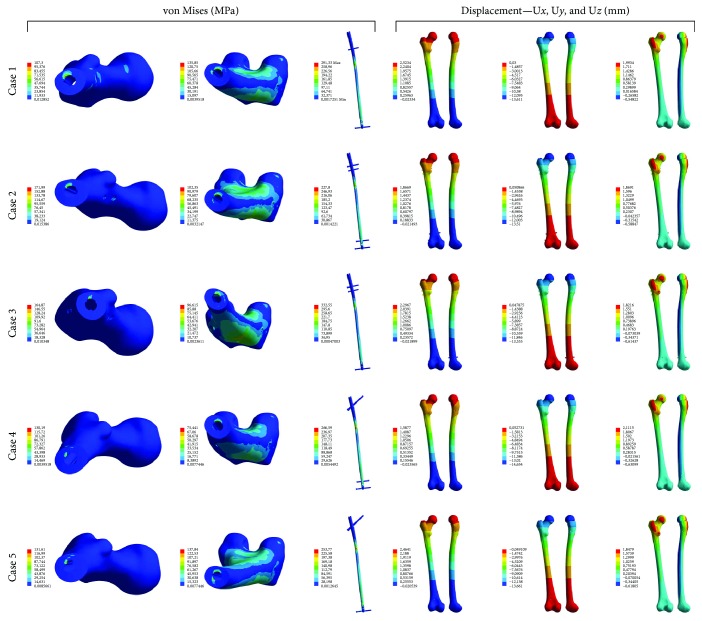
Results for analysed configurations of the stabilisation system for femoral diaphyseal fractures—state of stresses and displacements.

Results for all cases were presented in Figure 5.

**Case 1 .**

Displacements in the *x*-axis of the system with the maximum value of 2.52 mm indicated bone bending in the frontal plane and tilting of the proximal part of the bone to the side of the body. In the *y*-axis, there was a noticeable forward tilting of the proximal epiphysis with the maximum value of almost 13.6 mm. Displacements in the z-axis observed in the greater trochanter and bonehead corresponded to the direction of the axis, while the anterior part of the bone was displaced slightly in the opposite direction. Uneven distribution of isolines indicated slight rotational movements of the bone around the anatomical axis.

Reduced stresses observed in the bone peaked in the area of the fracture gap at the point of contact between the bone and the implant. The recorded maximum value was close to 135.85 MPa and was located at the medial edge of the hole below the fracture gap. Above the fracture gap, the recorded maximum stress was 107.3 MPa and was located at the posterior part of the medullary canal. The stresses in the implant material were located in the area of the fracture gap and amounted to 291.33 MPa.

**Case 2 .**

Displacements in all axes were characterized by distribution analogous to case 1. In the *x*-axis, the maximum value of displacements was slightly greater at 2.34 mm. In the *y*-axis, the value was 12.91 mm and the displacements were characterised by distribution analogous to case 1. A similar increase in the value of displacements with the preservation of their character was observed in the *z*-axis, where the maximum recorded value was 1.73 mm. The stresses observed in the area of contact of the implant with the bone tissue were characterised by a similar distribution—maximum stresses were located at the fracture edge on the wall of the medullary canal containing the nail; in particular, above the fracture gap, the value of stress in the bone tissue was 171.99 MPa, while below the fracture gap the value already decreased to nearly 102.35 MPa. The stressed recorded in the nail material reached a maximum of 277.8 MPa and were located in the area of the fracture gap.

**Case 3 .**

Case 3 is the first one where locking screws were inserted in the sagittal plane. The largest displacements in the *x*-axis occurred in the proximal part of the femur with the maximum value of 2.29 mm; there was a visible bending of the bone in the frontal plane and tilting of the proximal part of the bone to the side of the body. The largest displacements in the bone were shown by movements along the *y*-axis. Within the proximal part of the bone, the observed maximum absolute value was as high as 13.30 mm. The above displacements result in bending in the sagittal plane, and the proximal part of the bone becomes strongly tilted forward. The maximum value of displacements in the *z*-axis was 1.82 mm. The anterior part of the bone is displaced downwards, while within the lower trochanter as well as the posterior part of the greater trochanter and the head, there is a noticeable displacement upwards. The stresses recorded in the bone and on the walls of the medullary canal near the fracture gap amounted to 164.85 MPa and 96.64 MPa, respectively, above and below the damage site. The maximum stress recorded in the implant material was located, as in earlier cases, at the level of fracture gap and reached 332.55 MPa.

**Case 4 .**

The largest displacements in the *x*-axis occurred in the proximal part of the femur—on the femoral head, within the upper area of the neck, and in the area of insertion of the intramedullary nail—and their maximum value was 1.59 mm. The distribution map of displacements in the *x*-axis shows bone bending in the frontal plane and tilting of the proximal femoral epiphysis to the side of the body. Analysis of displacements in the *y*-axis, which is responsible for movement in the sagittal plane, showed the greatest values of displacements out of all three axes. Displacements were largest in the area of the head and at the place of nail insertion, with the maximum absolute value of 14.65 mm. The resulting displacements are caused by bending of the bone in the sagittal plane and show forward tilting of the proximal epiphysis and, to a large extent, of the diaphysis. The smallest displacement values were observed for the *z*-axis, where the maximum value was 2.11 mm. The area of greatest displacements in this axis was related to the small trochanter, the posterior part of the femoral head, and the posterior part of the greater trochanter. By far, most of the proximal femoral epiphysis moved upwards under the influence of positive displacements. However, distribution of displacements in this axis is not represented by evenly spaced bands, which indicates minimal rotational movements of the femur around the anatomical axis.

Stress in the bone material reached the maximum value at the edge below the fracture gap at the point of contact with the implant and amounted to almost 131 MPa. Above the fracture gap, a similar maximum already achieved a value nearly twice as low, amounting to 75.44 MPa. The maximum stress in the nail material was recorded at the point of contact with the fracture gap at the anterior site and amounted to 266.58 MPa.

**Case 5 .**

The maximum displacement in the *x*-axis was 2.46 mm. This illustrated bending of the bone in the frontal plane and tilting of the proximal part to the side of the body. The maximum displacement in the *y*-axis was as high as 13.66 mm. There was strong tilting of the proximal part of the bone and even bending, to a large extent, of the diaphysis towards the front of the femur. The smallest displacements were observed for the *z*-axis, where the maximum value was 1.85 mm. Distribution of isolines indicated minimal rotational movements of the femur around the anatomical axis.

The stresses in the bone tissue in the area of the fracture gap exceeded 130 MPa both above and below the fracture gap. In the case of implant, similarly as before, the maximum stress fluctuating around 254 MPa was observed at the height of the fracture gap.

### 3.1. Comparative Analysis of Displacements

**Figure 6:**
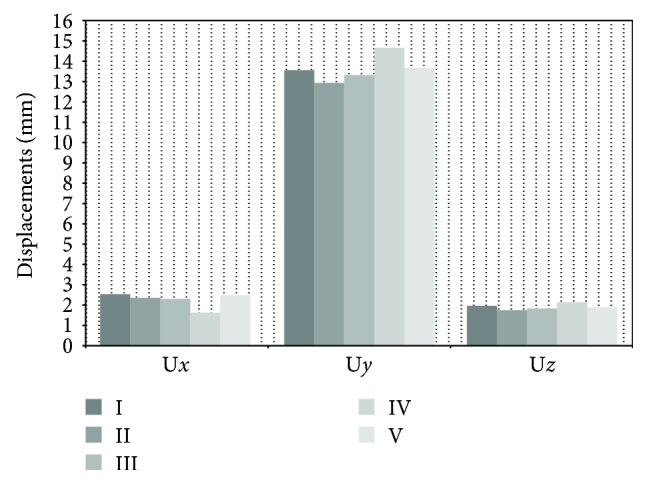
The maximum displacements obtained for every case for each axis of the reference system.

Comparative analysis of displacements (see Figure 6) in the *x*-axis showed that the differences in displacements in this axis are negligible except for Case 4. The highest maximum displacement of 2.52 mm occurred in Case 1, and the smallest one, at 1.59 mm, occurred in Case 4. The area of largest displacements was found on the femoral head, within the upper area of the neck, and around the area of insertion of the intramedullary nail into the bone. The distribution map showed bone bending in the frontal plane and tilting of the proximal part to the side of the body. Somewhat greater differences in displacements were found in the comparative analysis of the *y*-axis, in which the highest maximum of 14.65 mm occurred in Case 4, while the smallest one, at 12.91 mm, occurred in Case 2. Displacements of the order of 13 mm were also observed in Cases 1, 3, and 5. In this axis, too, the trends in displacement distribution were the same in all models; the observed bending in the sagittal plane resulted in strong tilting of the proximal femoral epiphysis and, to a large extent, bending of the diaphysis towards the front of the bone. The trend from the analysis of the *y*-axis was also visible in the *z*-axis—the highest maximum displacements in this axis were seen in Case 4, while the smallest ones were seen in Case 2. The differences in displacements for individual cases were negligible (in the order of millimetres). In all models, there was the same characteristic distribution of vertical bands arranged at a slight angle, which shows there were minimal rotational movements of the bone around the anatomical axis. In addition, the largest displacements were observed in the area of the small trochanter, the posterior part of the femoral head, and the posterior part of the greater trochanter.

### 3.2. Stress Analysis

**Figure 7:**
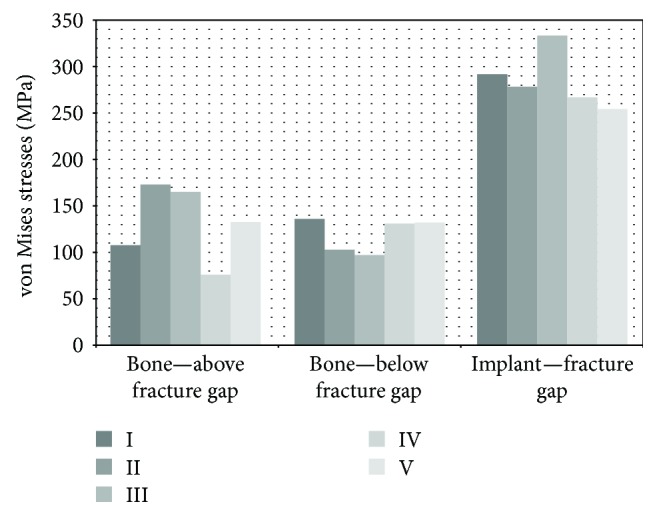
The maximum stresses obtained for every case at the points of concentration.

A comparison was made (see Figure 7) of the maximum stresses recorded at the points of concentration in the bone above the fracture gap at the point of contact with the callus and the nail. In all cases, such concentration occurred at the posterior part of the bone. This results from stretching of the bone while bending the proximal epiphysis in the sagittal plane towards the front of the bone. The differences in stress values are significant, with the highest values obtained in the case that reflected the compressive compression method (171.99 MPa), followed by smaller values in the case that used distal stabilisation in two planes (164.85 MPa), and somewhat smaller values (131.61 MPa) in the case that used a reconstruction screw and locking of the bottom part of the nail in the sagittal and frontal planes. The smallest maximum stress (75.44 MPa) in this area was recorded for the model that used a reconstruction screw and two screws in the distal part. This value is almost twice as low as the maximum cumulative stress in the bone above the fracture gap in Case 3. The classic case—107.03 MPa had stress values that put it between the obtained maximum values.

The comparison of cumulative stresses in the bone below the fracture gap at the point of contact with the callus, and the nail showed that the high values of stress on one side of the gap reduced concentration on the other side, with this trend being disturbed in Case 5. The classic case with the maximum value of 135.85 MPa did not differ significantly from Cases 4 and 5 with the values of, respectively, 130.8 MPa and 131.84 MPa. For Cases 2 and 3, the obtained values were 102.35 MPa and 96.64 MPa, respectively. In the cases where reconstruction screws were used in addition to locking screws, the concentration covered a larger area—on the medial and posterior sides. The observed concentration at the posterior part of the bone below the fracture gap is the result of stretching of the bone due to bending in the sagittal plane. On the other hand, the concentration on the medial part of the bone is the result of minimal rotation of the bone around the anatomical axis.

In all cases, stress concentrations were generated in the nail material in the area of the fracture gap. Slight concentrations also occurred near the holes through which the screws had been passed as well as in the areas of unused holes. Apart from Case 3, the maximum values for all cases ranged between 253.8 and 291.33 MPa. In Case 3, stress reached the maximum of 332.55 MPa. In each case, those values are well below the critical parameters of the titanium alloy used.

## 4. Discussion

A fracture of the type presented in this study is most often caused by a high-energy trauma and, therefore, primarily affects young people [25]. Despite the apparent lack of complexity, its treatment is not always successful [26]. The location of the femur in the kinematic chain and the resulting loads may, in combination with an improperly stabilised fracture, result in the formation of a pseudoarthrosis. In this study, it was assumed that the treated person was healthy with normal a bone tissue structure. It was also assumed that the applied load (half of the nominal value) was associated with protective lifestyle. However, it should be remembered that some of the key factors determining the possibility of using a particular solution include the condition of the bone tissue of the patient, which is especially important in the case of patients, mostly elderly, suffering from osteoporosis [27, 28] and the loads, which can exceed the safe values indicated by the medical staff. The stresses observed in the bone tissue in Cases 2 and 3 obtain values which, in the adopted loading model, should be considered potentially dangerous and could possibly lead to degradation of the bone material, which, in turn, could lead to a loss of stability of bone fragment fixation. Destabilisation of the system requires surgical treatment, which in turn raises the risk of treatment failure [16, 29].

Apart from the classic Case 1, the cases developed for the purposes of the analysis were paired in such way as to determine if the use of a neck reconstruction screw gives a significant advantage over static proximal stabilisation with two locking screws in the frontal plane. Case 2 was paired with Case 4 while Case 3 was paired with Case 5. Differences within each pair occurred only in the area of proximal stabilisation. Cases 2 and 4 represented distal stabilisation realized with two screws in the frontal plane, with one screw placed in the oblong hole and another one placed below it in the last oval hole of the nail. Cases 3 and 5 represented distal stabilisation realized with two screws, with one screw placed in the oval hole in the frontal plane and another one placed in the sagittal plane, also in the oval hole.

Cases 4 and 5, stabilised proximally with a reconstruction screw, give greater displacements along the *y*-axis and higher stresses in the bone below the fracture gap, but in relation to Case 3, those values can be considered less dangerous. In those cases, relatively high stresses below the fracture gap result in relatively small stresses on the other side of the fracture. In this comparison, Case 4 must be regarded as safest due to the lowest obtained stresses.

At the fracture level, stresses in the implant fall within the range of 230–288 MPa, which is well below the critical parameters of the material from which the implant was made. Therefore, it should be assumed that in the modelled system and for the selected maximum load, the mechanically weak link will be the tissue of the patient.

With regard to the obtained results, it can be concluded that the use of a greater number of support points and the distribution of the load over a greater volume of the femoral head by means of a reconstruction screw helps to advantageously reduce stress concentrations in the implant and diminishes the risk of its damage and the resultant destabilisation of bone fragments. Compared to the classic case, the use of a reconstruction screw and more comprehensive distal stabilisation allowed to reduce the stresses in the implant itself by 24 MPa in Case 4 and by 37 MPa in Case 5. The use of a distal two-plane stabilisation worsened the stress conditions in the fracture gap, but they still fall within the boundaries set by the classic case.

The choice of the appropriate type of stabilisation is one of the critical factors that largely determine the success of the treatment [30, 31]. The ever increasing computing capabilities in conjunction with the increasingly accurate imaging methods provide tremendous opportunities in the numerical representation of the implant-bone system. Clinical practice combined with numerical calculations is a powerful prediction tool that makes it possible to indicate the most advantageous stabilisation variant in a given case [32], identify rehabilitation opportunities [33], and so forth.

## 5. Conclusions

Combining practical clinical experience with theoretical numerical models not only allows clinicians to enhance the diagnosis of the case and take the right steps but also increases the ability to generate models more tailored to the actual parameters of the patient. The limitations that arise at modelling and defining boundary conditions mean that the resultant model has a certain margin of error; nevertheless, they allow to indicate general trends that may be very helpful in clinical work. Ultimately, the obtained results indicate that there is a visible advantage to one-plane distal stabilisation in the reduction of stresses regardless of the type of proximal stabilisation; by contrast, two-plane distal stabilisation worsens stress conditions in the fracture gap. The last conclusion pertains to the possibility of damage to the implant—the results of calculations indicate that the use of proximal stabilisation with a neck screw reduces such risk.

The limitations of this study include the adopted simplifications at the level of the geometric model—the radii of the edge curvatures of the implant holes were not taken into account, and the presence of threads was omitted in the case of bone screw modelling; however, the latter limitation seems irrelevant if we assume that the interface between the bone screw and the bone and between screw and implant is stable and does not disconnect. This is also a simplification because in a real structure we observe much more complicated contact phenomena which limited accuracy of the calculations. The isotropic and linear mechanical properties of the modelled bone-implant system were also adopted. The model also ignores the presence of the surrounding soft tissues, which have a natural tendency to limit the mobility of bone fragments. The last implemented simplification was in the fracture gap where any processes of remodeling were not taken into account. Presented simulation relates to the early days after fracture stabilization. This design decision was dictated by clinical experience, which allows the authors to assume that taking into account the presence of such tissues could reduce the intensity of nodal displacements in the model.

## Figures and Tables

**Table 1:** Loading forces at the proximal femoral epiphysis during one-leg stance.

	*F_x_* [*N*]	*F_y_* [*N*]	*F_z_* [*N*]
*R*	245	−35	−651
*M*_a_	−44	−11.5	33
*M*_b_	−151.5	−101	109
*T*	−20.5	−7.5	0
*R*_u_	−21	−178.5	165

**Table 2:** Material properties of the model [1, 23, 24].

Material	Material properties	
	E [MPa]	ν [−]
Compact bone	16.700	0.3
Cancellous bone	155	0.3
Fracture gap tissue	2	0.4
Ti6Al7Nb	105.000	0.36

## Abbreviations

AO Foundation: The AO Foundation is a medically guided, not-for-profit organization led by an international group of surgeons specialized in the treatment of trauma and disorders of the musculoskeletal system (according to aofoundation.org)
CT: Computed tomography.
